# Predisposing factors to the practice of self-medication in Brazil: Results from the National Survey on Access, Use and Promotion of Rational Use of Medicines (PNAUM)

**DOI:** 10.1371/journal.pone.0189098

**Published:** 2017-12-08

**Authors:** Emilia da Silva Pons, Daniela Riva Knauth, Álvaro Vigo, PNAUM Research Group, Sotero Serrate Mengue

**Affiliations:** Graduate Program in Epidemiology, Federal University of Rio Grande do Sul, Porto Alegre, Rio Grande do Sul, Brazil; The Chinese University of Hong Kong, HONG KONG

## Abstract

**Objective:**

To understand the predisposing factors that lead to the practice of self-medication and the factors associated with the use of medicines via self-medication in the adult population of Brazil.

**Methods:**

The analyzed data are part of the National Survey on Access, Use and Promotion of Rational Use of Medicines (PNAUM), a survey whose population consisted of individual residents permanently domiciled in urban areas in Brazil. In this work, the data references the 31 573 respondents aged 20 or higher (76.2% of the final PNAUM sample). Poisson regression models with robust variance were used for estimating the independent effect of each variable with medicine use via self-medication.

**Results:**

Of the interviewees, 73.6% stated they had used some medication without medical recommendation if they had previously used the same product; 73.8% stated they had used non-prescribed medicine when the medicine was already present at home; and 35.5% stated they had used some non-prescribed medication when they knew someone who had already taken the same medication. The prevalence of self-medication was 18.3%. The variables associated with the highest probability of using medicine via self-medication were: geographic region within Brazil, gender, age group, per capita income, self-assessment of health, self-reported use of previously used non-prescribed medication, and self-reported use of non-prescribed medication when that medication was already present at home.

**Conclusions:**

The use of medicines via self-medication in Brazil is relatively frequent and influenced by previous experience and familiarity with the medications, and is more common among women and individuals with low self-assessment of health.

## Introduction

The World Health Organization (WHO) defines self-medication as the selection and use of medicines by individuals to treat self-recognized illnesses or symptoms, and thus, self-medication is one element of self-care [1–3]. To some authors, however, self-medication is the acquisition and consumption of one or more medications without medical counseling [4,5]. In Brazil, according to current law, self-medication is defined as the use of medication without a prescription, guidance, and/or the supervision of a physician or dentist [6].

Self-medication takes various forms and the literature has shown that it is not limited to acquisition of non-prescribed medications. Indeed, the practice of self-medication extends through the use of leftover prescription medications and the sharing of medications with members of one’s family and social circle [7] to the reutilization of old prescriptions [8] and the altering of the dosage of prescribed medications [7,9].

Obtaining precise estimates of self-medication comprises a difficult task, as it deals with a practice that lies in the sphere of private activities and/or is subject to judgment. Beyond this, the lack of uniform methodologies among studies and the fact that many of them consist of samples circumscribed to restricted populations (such as the elderly, the pregnant, or health care professionals) contributes to the difficulty in obtaining said estimates, which in turn leads to quite varied prevalence results. In international studies, the prevalence of self-medication range from 12 to 90% [7,10–17].

In Brazil, studies on the prevalence and factors associated with self-medication are rare and most involved samples limited to a single municipality or a few small-sized municipalities. In two small municipalities in southern Bahia State, the prevalence of self-medication was verified as 74% [18]; in a medium-sized municipality in the state of Rio Grande do Sul, the prevalence of self-medication was 53.3% [8]; and in one cross-sectional study undertaken in a municipality in the state of Minas Gerais, 28% of the participants exclusively consumed medicines not prescribed by physicians. In this last study, gender, age, household size, number of medical consultations in the last 12 months, if a pharmacist has been consulted in the last 12 months, and money spent on medications in the last 12 months were all shown to be associated with the exclusive consumption of non-prescribed medications [19]. Another study performed in pharmacies in three Brazilian states showed that the choice of medication for self-medication is based primarily on the recommendation of lay people (51%) and on the influence of prior prescriptions (40%) [20]. Finally, in a study done in 2003 with 5 000 adults (World Health Survey–WHS), 25% of the individuals who used medications in the 15 days prior to the interview did so without a prescription from a health care professional [21].

Hence, the objective of this study was to understand the predisposing factors that lead to the practice of self-medication and the factors associated with the use of medicines via self-medication in the adult Brazilian population. We understand the predisposing factors as elements present in the daily practices and conceptions of a particular social group that guide certain behaviors, in the present case, the use of non-prescribed medications. Our hypothesis is that the individuals activate different evaluation criteria in the decision to use non-prescribed medications.

## Materials and methods

The present study is based on data collected by the National Survey on Access, Use and Promotion of Rational Use of Medicines (PNAUM, Pesquisa Nacional sobre Acesso, Utilização e Promoção do Uso Racional de Medicamentos no Brasil), carried out between September, 2013 and January, 2014. It is a survey with a probabilistic sample in three stages, in which the primary sampling unit corresponds to the municipalities, the second stage to the census sectors (as defined by the 2010 Brazilian Census carried out by the Brazilian Institute of Geography and Statistics), and the third to domiciles. The study population were residents of permanent, private domiciles in the urban areas of Brazil. The data collection strategy utilized was the face-to-face interview. In the end, a sample of 41 533 people was extended to represent the urban Brazilian population registered by the 2010 Census. The data analyzed in this work has been limited to the 31 573 individuals aged 20 or higher who responded to the PNAUM, which represents 76.2% of the sample. The extension of the sample and the complexity of the sampling plan for representing the relevant urban Brazilian population age-group were taken into account with each analysis performed. Additional methodological details of the PNAUM are described by Mengue et al. [22].

Self-medication was categorized two ways:

1. The use of medicines through self-medication: all the interviewees that used any medication, excluding contraceptives, in the 15 days preceding the interview without a recommendation by a physician or dentist were considered users of medicine via self-medication. This was the variable used in both the calculation of the self-medication prevalence and as the outcome of the multivariable analysis.
2. Predisposing factors to the practice of self-medication: the predispositions of individuals to practice self-medication were explored through the following questions: (a) Do you take a non-prescribed medication if the medication is available at home?; (b) Do you take a non-prescribed medication if you know someone who has taken it before?; (c) Do you take a non-prescribed medication if you have taken the medication before?; (d) Do you take a non-prescribed medication after reading the instructions or other information?; (e) Do you take a non-prescribed medication if you can get it easily?. The respondent had three options for responding to the questions: “yes”, “no”, or “I declare that I do not use non-prescribed medication”.

In order to analyze the predisposing and associated factors related to the use of medicines via self-medication, a Poisson regression model with robust variance fitted. The dependent variable was the use of medicine via self-medication (yes or no). The independent variables tested were: geographic region of Brazil, gender, age-group, marital status, schooling, per capita income, self-assessment of health, presence of chronic disease, declaration of non-prescribed medication use based on previous use of the same medication, declaration of use of non-prescribed medication when the medication was already present at home, and declaration of non-prescribed medication use based on knowing someone who had already used the same medicine. Only three of the five variables related to the predisposing factors for self-medication were included in the model, as we understood these to be directly related to the greater familiarity of individuals with certain medications, the central hypothesis of the present article. Possible interactions between these variables related to the predisposing factors for self-medication were also considered and tested in the model.

In all the described analyses, the categorical variables were represented by relative frequencies accompanied by their respective 95% confidence intervals. The relative frequencies presented are weighted according to sample weights.

The project was approved by the Comissão Nacional de Ética em Pesquisa (National Commission for Ethics in Research). All the participants signed two copies of the consent form before responding to the interview.

## Results

Sociodemographic characteristics, information about health and prevalence of self-medication in the adult Brazilian population studied by PNAUM are presented in Table 1. The sample was composed predominantly by women (53.8%). Three-fourths of the interviewees reported themselves as being in good or very good health and 39.1% suffered from a chronic disease. The prevalence of use of medicine via self-medication was 18.3%.

**Table 1:** Sociodemographic characteristics, information about health and prevalence of use of medicines via self-medication in the adult population of Brazil studied by PNAUM. PNAUM, Brazil, 2014.

Characteristic	Prevalence^a^ (%)	95% CI
**Region of Brazil**		
North	6.7	5.3–8.5
Northeast	23.1	19.1–27.8
Southeast	47.7	41.8–53.6
South	14.7	11.8–18.1
Central-west	7.8	6.1–9.9
**Gender**		
Male	46.2	45.1–47.3
Female	53.8	52.7–54.9
**Age group**		
20 a 29	23.8	22.5–25.1
30 a 39	21.9	20.8–23.1
40 a 49	19.8	18.8–20.8
50 a 59	16.4	15.6–17.2
60 a 69	9.8	9.3–10.4
≥ 70	8.3	7.7–8.9
**Race**		
White	46.7	44.0–49.3
Brown	9.6	8.6–10.6
Asian	1.2	1.0–1.5
Mulatto	42.1	39.9–44.5
Indigenous	0.4	0.3–0.6
**Marital Status**		
Living with partner	61.5	60.2–62.8
Not living with partner, but used to	20.3	19.4–21.3
Never lived with a partner	18.2	16.9–19.5
**Education (years completed)**		
0 to 8	57.7	56.0–59.3
9 to 11	31.0	29.7–32.3
≥ 12	11.3	10.3–12.4
**Per capita income (quartiles)**		
≤ US$ 100.00	17.7	15.8–19.8
US$ 100.01 a US$ 200.00	27.0	25.3–28.8
US$ 200.01 a US$ 300.00	20.8	19.5–22.2
≥ US$ 300.00	34.5	31.9–37.2
**Self-assessment of health**		
Very poor/poor	3.4	3.0–3.7
Regular	22.1	20.8–23.4
Good	56.5	55.2–57.7
Very good	18.1	16.7–19.5
**Presence of chronic disease**	39.1	37.8–40.5
**Use of medicines via self-medication**	18.3	16.9–19.9

^a^ n = 117 761 374.

Percentages weighted by sample weights

Of the interviewees, 32.6% stated that they never make use of non-prescribed medications. Of these, 51.1% were male, 60.1% had completed at least 8 years of education, 60.7% lived with a partner, and 81.1% reported their health as good or very good.

Table 2 presents the relative frequencies of the predisposing factors to the practice of self-medication. The prevalences of using medicine via self-medication according to sociodemographic characteristics, health profile, and predisposing factors to the practice of self-medication are presented in Table 3, together with the crude prevalence ratios (PR) and their respective 95% confidence intervals. Note a significant increase in the prevalence of self-medication among individuals who report their health as average or poor/very poor, with crude prevalence ratios PR = 1.88 (95% CI, 1.59–2.22) and PR = 1.91 (95% CI, 1.55–2.35) respectively. The crude PR among the individuals who stated using non-prescribed medication when it was already present in the home (PR = 2.35; 95% CI, 2.03–2.73) or when they had previously used the medication (PR = 2.47; 95% CI, 2.14–2.85) are also notable.

**Table 2:** Prevalence^a^ of predisposing factors to the practice of self-medication in the adult population of Brazil studied by PNAUM. PNAUM, Brazil, 2014.

Predisposing factors	Prevalence (%)	95% CI
Uses non-prescribed medication based on having previously used the same medication	73.6	71.5–75.6
Uses non-prescribed medication based on availability in the home	73.8	72.0–75.5
Uses non-prescribed medication based on knowing someone who had already used the same medicine	35.5	33.6–37.3
Uses non-prescribed medication if has easy access to it	20.0	18.5–21.6
Uses non-prescribed medication after having read the instructions	32.1	30.1–34.1

^a^ n = 79 145 706 (individuals who related engaging in at least one of the predisposing factors to the practice of self-medication).

Percentages weighted by sample weights.

**Table 3:** Prevalence and crude prevalence ratios for the use of medicines via self-medication in the adult population of Brazil studied by PNAUM, according to the sociodemographic and economic characteristics, health profile, and predisposing factors to the practice of self-medication. PNAUM, Brazil, 2014.

Characteristic	Prevalence^a^ (%)	95% CI	*p*-value^b^	Crude PR^c^	95% CI	*p*-value
**Region of Brazil**			**<0.001**			**<0.001**
Southeast	14.8	12.7–17.3		1	–	
North	20.1	17.0–23.5		1.35	1.08–1.69	
Northeast	27.0	24.4–29.9		1.82	1.51–2.19	
South	13.7	12.2–15.4		0.92	0.76–1.12	
Central-West	21.0	19.1–23.0		1.41	1.18–1.70	
**Gender**			**<0.001**			**<0.001**
Male	15.1	13.4–17.0		1	–	
Female	21.1	19.5–22.8		1.39	1.25–1.55	
**Age group**			**<0.001**			**<0.001**
20 to 29	19.7	17.5–22.2		1	–	
30 to 39	20.9	18.5–23.6		1.06	0.93–1.21	
40 to 49	19.1	17.3–21.1		0.97	0.86–1.09	
50 to 59	16.1	14.3–18.2		0.82	0.72–0.93	
60 to 69	15.0	13.4–16.8		0.76	0.66–0.87	
≥ 70	13.9	12.0–16.0		0.70	0.60–0.82	
**Marital Status**			**0.288**			**0.271**
Living with partner	19.1	17.6–20.8		1	–	
Not living with partner, but used to	20.3	18.4–22.4		1.06	0.97–1.67	
Never lived with a partner	18.3	15.7–21.2		0.95	0.84–1.08	
**Education (years completed)**			**0.587**			**0.588**
0 to 8	18.1	16.5–19.8		1	–	
9 to 11	18.1	16.4–20.0		1.00	0.91–1.10	
≥ 12	19.5	16.7–22.5		1.07	0.93–1.24	
**Per capita income (quartiles)**			**<0.001**			**<0.001**
≤ US$ 100.00	29.0	25.8–32.4		1	–	
US$ 100.01 to US$ 200.00	22.1	19.8–24.5		0.76	0.68–0.86	
US$ 200.01 to US$ 300.00	20.5	17.9–23.5		0.71	0.60–0.83	
≥ US$ 300.00	22.3	20.1–24.6		0.77	0.66–0.89	
**Self-assessment of health**			**<0.001**			**<0.001**
Very good	13.5	11.4–15.9		1	–	
Good	16.7	15.3–18.2		1.24	1.07–1.43	
Regular	25.4	23.1–27.8		1.88	1.59–2.22	
Poor/Very poor	25.8	22.4–29.4		1.91	1.55–2.35	
**Presence of chronic disease**			**<0.001**			**<0.001**
No	17.2	15.7–18.8		1	–	
Yes	20.1	18.4–22.0		1.17	1.08–1.27	
**Uses non-prescribed medication when the medication is already present at home**			**<0.001**			**<0.001**
No	12.3	10.5–14.3		1	–	
Yes	28.9	27.0–30.8		2.35	2.03–2.73	
**Uses non-prescribed medication based on knowing someone who had already used the same medicine**			**<0.001**			**<0.001**
No	19.9	18.2–21.7		1	–	
Yes	32.9	30.6–35.3		1.66	1.51–1.82	
**Uses non-prescribed medication based on having previously used the same medication**			**<0.001**			**<0.001**
No	11.8	10.2–13.5		1	–	
Yes	29.1	27.2–31.0		2.47	2.14–2.85	

^a^ Percentages weighted by sample weights.

^b^ Pearson's χ^2^-test.

^c^ Poisson regression with robust variance.

The results of the multivariable analysis are presented in Table 4. The variables that revealed themselves as significantly associated with a higher probability of use of medicines via self-medication were: geographic region of Brazil, gender, age group, per capita income, self-assessment of health, declaration of non-prescribed medication use when the medication is already present at home, and declaration of non-prescribed medication use if the individual had previously used the same medication. The interaction between “Uses non-prescribed medication when the medication is already present at home” and “Uses non-prescribed medication based on having previously used the same medication” was shown to be statistically significant.

**Table 4:** Adjusted prevalence ratios for the use of medicines via self-medication in the adult population of Brazil studied by PNAUM, according to the sociodemographic and economic characteristics, health profile, predisposing factors to the practice of self-medication, and interaction test between the variables related to the predisposing factors to the practice of self-medication. PNAUM, Brazil, 2014.

Characteristic	Adjusted PR	95% CI	*p*-value^a^
**Region of Brazil**			**<0.001**
Southeast	1	–	
North	1.14	0.93–1.41	
Northeast	1.41	1.20–1.65	
South	0.82	0.68–0.98	
Central-West	1.22	1.04–1.44	
**Gender**			**0.007**
Male	1	–	
Female	1.21	1.05–1.39	
**Age group**			**0.011**
20 to 29	1	–	
30 to 39	1.02	0.87–1.20	
40 to 49	1.02	0.88–1.18	
50 to 59	0.84	0.71–0.99	
60 to 69	0.81	0.69–0.97	
≥ 70	0.82	0.66–1.00	
**Per capita income (quartiles)**			**0.012**
≤ US$ 100.00	1	–	
US$ 100.01 to US$ 200.00	0.87	0.77–0.98	
US$ 200.01 to US$ 300.00	0.91	0.77–1.08	
≥ US$ 300.00	1.06	0.92–1.22	
**Health self-evaluation**			**<0.001**
Very good	1	–	
Good	1.19	0.99–1.42	
Regular	1.44	1.19–1.73	
Poor/Very poor	1.42	1.12–1.80	
**Uses non-prescribed medication when the medication is already present at home (A)**			**<0.001**
No	1	–	
Yes	2.19	1.62–2.90	
**Uses non-prescribed medication based on having previously used the same medication (C)**			**<0.001**
No	1	–	
Yes	2.38	1.77–3.19	
**Interaction:**			
(A)*(C)	0.61	0.42–0.88	**0.009**

^a^ Poisson regression with robust variance.

## Discussion

Previous experiences with medications and availability of them at home are factors that predispose individuals their use via self-medication. These experiences can be either of one’s own use or that of people close to the self-medicator. In this study, 73.6% of the interviewees stated they had used some medication without a physician’s recommendation if they previously used the same product, and 73.8% stated they had used a non-prescribed medication if the medication was available in the home. Additionally, 35.5% of the participants declared using some non-prescribed medication if they knew someone who had taken the same medication. These factors favored the actual use of non-prescribed medications. The associations discovered indicate that the criteria of user familiarity with the medications are activated in the decision to use a medication without a prescription from a medical professional. Self-medication with medicines previously prescribed or recommended by friends or family appears in other studies. In one study of 5 251 people who bought non-prescribed medication in pharmacies in Latin America, 82% of the medications were purchased more than once, 42% of all medications were bought based on a previous medical recommendation, and 28% on a recommendation from a family member or friend [23]. In another study of 309 residents of the Lisbon metropolitan area, 63.8% of the medications used the last time the interviewee self-medicated had been previously prescribed by a physician or recommended by a pharmacist. According to the author of the aforementioned study, the medications selected by users for use via self-medication are, in general, those they have already proven, through their own experience, to produce the desired results [24]. In our study, beyond experience, having the medication at home or easily accessible is also a factor which influences self-medication.

The use of medicines via self-medication, however, happens in a context where the user possesses some reference, be it direct, through own experience, or indirect, though that of those close to him or her, to the medication being used. This allows the user to make a lay evaluation of the benefits and risks involved in the option of self-medication. According to Lopes [25], familiarity with a medication can be understood as a form of routinization which allows for a reduction of unpredictability and uncertainty in its results, thus engendering practical confidence and functioning as a safeguard in the face of possible risks. Beyond that, through social relationships, descriptions of good or bad experiences using the medication are spread and pass to create a reference system accessible for validation via direct empirical experience. Fainzang [26] goes further still in stating that, although self-medication implies personal choice, individuals are never completely solitary and independent in this choice, as they are subjected to not only the influence of others around then, but also to that of society as a whole.

One other context that conveys the importance of familiarity with a medicine for self-medication is that of chronic disease. In our study, we found an association between use of medicine via self-medication and chronic disease in the unadjusted model, but after adjustment that association became statistically non-significant. The chronically ill tend to have greater familiarity with medications and this familiarity can lead them to greater consumption of medicines via self-medication. On the other hand, as the chronically ill tend to monitor their heath more regularly, it is possible that self-medication done by these patients occurs not only through the acquisition of non-prescribed medications but also (or only) through alteration of recommended dosages. These posological changes of prescribed medications could be considered as self-medication if a broader definition were adopted. Association between self-medication and chronic disease is also observed in previous studies [15,27].

Another element contributing to the use of medicines via self-medication is the individuals’ perception and degree of tolerance for sensations of morbidity. “Pharmaceutical consumption” without a prescription is, according to Boltanski [28], one of the indicators of this perception, denoted by the author as “medical need”. In this context, and in light of the findings of this study, people whose self-assessment of heath is low (regular or poor/very poor) and who have, thus, a negative perception of their health and a low tolerance for physical signs and symptoms, tend to resort to self-medication as a strategy for fulfilling an identified “medical need”. This association between self-assessment of health and use of non-prescribed medication also appears in a study done in Denmark [27]. Even within the context of “medical need”, it was noted that younger individuals (20 to 49 years old) resort more often to self-medication, indicating a lower tolerance for physical signs and symptoms. The same behavior was observed in the previously mentioned study by Nielsen et al. [27], where respondents between the ages of 25 and 44 years were more likely to use over-the-counter medication. One possible explanation for this is the fact that these individuals are, in general, economically active and have more acute events, necessitating a quick resolution of their problems. Older individuals, who tend to have more chronic diseases, are consequently more accustomed to a set of signs and symptoms related to their health conditions, but might not have the same urgency, and may therefore opt to seek a prescription.

The more frequent use of medicines via self-medication among women has been well-described in the literature [11,12,14,15,27] and was also found in the present study. Among the factors suggested to explain this behavior, some authors cite the higher usage of health services by women and their greater tendency for self-care [11,29]. Beyond this, women seem more alert than men to signs of illness, listening more to their bodies and more frequently maintaining a sensitive relationship with it [28]. Another hypothesis is that the greater medicalization of the female body contributes to less resistance by women to the use of medication [30–32].

The association between higher per capita income and the use of non-prescribed medications observed in our study could be explained by the greater economic ability to acquire medication by people in the top income quartile. Additionally, it may also be the case that users with low per capita income make greater use of public health services and thus obtain their medication within the Sistema Único de Saúde (SUS, the Unified Health Service) through medical prescription. In Brazil, all medications dispensed within SUS require a prescription.

The prevalence of the use of non-prescribed medicines via self-medication found in this study (18.3%) is quite close to the value found for the adult population of Spain (18.1%) coming from a study that covered a period of two weeks prior to the interview [14]. This prevalence, however, is lower than the values observed in other base population studies carried out in Brazil, whose prevalences ranged from 28% to 74% [8,18,19].

In conclusion, the use of medicines via self-medication in Brazil is relatively frequent and influenced by previous experience and familiarity with the medication. It is more prevalent among women and individuals with low self-assessment of health. Self-medication presents risks and benefits; nonetheless, as the data indicate, people in general engage in self-medication starting with some knowledge of the medication being used. This lay evaluation permits the user to identify the potential risks of the medications, as well as their benefits. In this sense, self-medication awareness campaigns that emphasize only the risks of the practice, as most have in Brazil, contribute little to safer use of medications inasmuch as, as the data show, a great portion of the population continues to resort to self-medication starting from their own criteria of familiarity with the medicine. Therefore, we suggest that self-medication awareness campaigns take these finding into consideration.

## Supporting information

S1 File PNAUM Self-medication database (XLS). (XLS)
